# Supplementary material for: Bayesian refinement of protein structures and ensembles against SAXS data using molecular dynamics
Source: PLoS Comput Biol. 2017 Oct 18;13(10):e1005800. doi: 10.1371/journal.pcbi.1005800 (PMC5662244; doi:10.1371/journal.pcbi.1005800)
Supplement: S1 Archive — (BZ2) [file pcbi.1005800.s011.tar.bz2 › gromacs_4.62_bayesian_saxs_refi/share/html/online/mdp.html]

mdp file format


## mdp file format

|  |  |
| --- | --- |
| Main Table of Contents | **VERSION 4.6** |
| GROMACS homepage | **Sat 19 Jan 2013** |

---

Follow this link for a detailed description of the options.

Below is a sample mdp file.
The ordering of the items is not important, but if you enter the same
thing twice, the **last** is used (grompp gives you a note when
overriding values). Dashes and underscores on the
left hand side are ignored.

The values of the options are reasonable values for a 1 nanosecond
MD run of a protein in a box of water.

---

```
title                    = Yo
cpp                      = /lib/cpp
include                  = -I../top
define                   = 
integrator               = md
dt                       = 0.002
nsteps                   = 500000
nstxout                  = 5000
nstvout                  = 5000
nstlog                   = 5000
nstenergy                = 250
nstxtcout                = 250
xtc-grps                 = Protein
energygrps               = Protein  SOL
nstlist                  = 10
ns-type                  = grid
rlist                    = 0.8
coulombtype              = cut-off
rcoulomb                 = 1.4
rvdw                     = 0.8
tcoupl                   = Berendsen
tc-grps                  = Protein      SOL
tau-t                    = 0.1  0.1
ref-t                    = 300  300
Pcoupl                   = Berendsen
tau-p                    = 1.0
compressibility          = 4.5e-5
ref-p                    = 1.0
gen-vel                  = yes
gen-temp                 = 300
gen-seed                 = 173529
constraints              = all-bonds
```

---

With this input grompp will produce
an mdout.mdp with all the options and descriptions:

---

```
; VARIOUS PREPROCESSING OPTIONS = 
title                    = Yo
cpp                      = /lib/cpp
include                  = -I../top
define                   = 

; RUN CONTROL PARAMETERS = 
integrator               = md
; start time and timestep in ps = 
tinit                    = 0
dt                       = 0.002
nsteps                   = 500000
; number of steps for center of mass motion removal = 
nstcomm                  = 1
comm-grps                = 

; LANGEVIN DYNAMICS OPTIONS = 
; Temperature, friction coefficient (amu/ps) and random seed = 
bd-temp                  = 300
bd-fric                  = 0
ld-seed                  = 1993

; ENERGY MINIMIZATION OPTIONS = 
; Force tolerance and initial step-size = 
emtol                    = 100
emstep                   = 0.01
; Max number of iterations in relax-shells = 
niter                    = 20
; Frequency of steepest descents steps when doing CG = 
nstcgsteep               = 1000

; OUTPUT CONTROL OPTIONS = 
; Output frequency for coords (x), velocities (v) and forces (f) = 
nstxout                  = 5000
nstvout                  = 5000
nstfout                  = 0
; Output frequency for energies to log file and energy file = 
nstlog                   = 5000
nstenergy                = 250
; Output frequency and precision for xtc file = 
nstxtcout                = 250
xtc-precision            = 1000
; This selects the subset of atoms for the xtc file. You can = 
; select multiple groups. By default all atoms will be written. = 
xtc-grps                 = Protein
; Selection of energy groups = 
energygrps               = Protein  SOL

; NEIGHBORSEARCHING PARAMETERS = 
; nblist update frequency = 
nstlist                  = 10
; ns algorithm (simple or grid) = 
ns-type                  = grid
; Periodic boundary conditions: xyz or none = 
pbc                      = xyz
; nblist cut-off         = 
rlist                    = 0.8
domain-decomposition     = no

; OPTIONS FOR ELECTROSTATICS AND VDW = 
; Method for doing electrostatics = 
coulombtype              = cut-off
rcoulomb-switch          = 0
rcoulomb                 = 1.4
; Dielectric constant (DC) for cut-off or DC of reaction field = 
epsilon-r                = 1
; Method for doing Van der Waals = 
vdw-type                 = Cut-off
; cut-off lengths        = 
rvdw-switch              = 0
rvdw                     = 0.8
; Apply long range dispersion corrections for Energy and Pressure = 
DispCorr                 = No
; Spacing for the PME/PPPM FFT grid = 
fourierspacing           = 0.12
; FFT grid size, when a value is 0 fourierspacing will be used = 
fourier-nx               = 0
fourier-ny               = 0
fourier-nz               = 0
; EWALD/PME/PPPM parameters = 
pme-order                = 4
ewald-rtol               = 1e-05
epsilon-surface          = 0
optimize-fft             = no

; OPTIONS FOR WEAK COUPLING ALGORITHMS = 
; Temperature coupling   = 
tcoupl                   = Berendsen
; Groups to couple separately = 
tc-grps                  = Protein      SOL
; Time constant (ps) and reference temperature (K) = 
tau-t                    = 0.1  0.1
ref-t                    = 300  300
; Pressure coupling      = 
Pcoupl                   = Berendsen
Pcoupltype               = Isotropic
; Time constant (ps), compressibility (1/bar) and reference P (bar) = 
tau-p                    = 1.0
compressibility          = 4.5e-5
ref-p                    = 1.0

; SIMULATED ANNEALING CONTROL = 
annealing                = no
; Time at which temperature should be zero (ps) = 
zero-temp-time           = 0

; GENERATE VELOCITIES FOR STARTUP RUN = 
gen-vel                  = yes
gen-temp                 = 300
gen-seed                 = 173529

; OPTIONS FOR BONDS     = 
constraints              = all-bonds
; Type of constraint algorithm = 
constraint-algorithm     = Lincs
; Do not constrain the start configuration = 
unconstrained-start      = no
; Relative tolerance of shake = 
shake-tol                = 0.0001
; Highest order in the expansion of the constraint coupling matrix = 
lincs-order              = 4
; Lincs will write a warning to the stderr if in one step a bond = 
; rotates over more degrees than = 
lincs-warnangle          = 30
; Convert harmonic bonds to morse potentials = 
morse                    = no

; NMR refinement stuff  = 
; Distance restraints type: No, Simple or Ensemble = 
disre                    = No
; Force weighting of pairs in one distance restraint: Equal or Conservative = 
disre-weighting          = Equal
; Use sqrt of the time averaged times the instantaneous violation = 
disre-mixed              = no
disre-fc                 = 1000
disre-tau                = 0
; Output frequency for pair distances to energy file = 
nstdisreout              = 100

; Free energy control stuff = 
free-energy              = no
init-lambda              = 0
delta-lambda             = 0
sc-alpha                 = 0
sc-sigma                 = 0.3

; Non-equilibrium MD stuff = 
acc-grps                 = 
accelerate               = 
freezegrps               = 
freezedim                = 
cos-acceleration         = 0
energygrp-excl           =

; Electric fields       = 
; Format is number of terms (int) and for all terms an amplitude (real) = 
; and a phase angle (real) = 
E-x                      = 
E-xt                     = 
E-y                      = 
E-yt                     = 
E-z                      = 
E-zt                     = 

; User defined thingies = 
user1-grps               = 
user2-grps               = 
userint1                 = 0
userint2                 = 0
userint3                 = 0
userint4                 = 0
userreal1                = 0
userreal2                = 0
userreal3                = 0
userreal4                = 0
```

---

http://www.gromacs.org
